# Supplementary material for: Immuno-Transcriptomic Profiling of Blood and Tumor Tissue Identifies Gene Signatures Associated with Immunotherapy Response in Metastatic Bladder Cancer
Source: Cancers (Basel). 2024 Jan 19;16(2):433. doi: 10.3390/cancers16020433 (PMC10814931; doi:10.3390/cancers16020433)
Supplement: Supplementary file 1 [file cancers-16-00433-s001.zip › cancers-2711662-supplementary.pdf]

Table S1. Blood All differential genes

ID	symbol	logFC_edger	logFC_deseq	logFC_voom	pvalue_edger	pvalue_deseq	pvalue_voom
ENSMUSG00000000157	Itgb2l	2,810	0,129	3,014	0,006	0,012	0,004
ENSMUSG00000000486	Sept1	0,649	0,244	0,608	0,004	0,004	0,008
ENSMUSG00000000903	Vpreb3	-1,915	-1,442	-1,818	0,001	0,000	0,006
ENSMUSG00000001248	Gramd1a	-0,600	-0,233	-0,609	0,006	0,005	0,004
ENSMUSG00000001323	Srr	0,747	0,166	0,782	0,004	0,015	0,005
ENSMUSG00000001424	Snd1	0,461	0,168	0,462	0,007	0,021	0,007
ENSMUSG00000001576	Ergic1	-0,869	-0,394	-0,797	0,002	0,001	0,003
ENSMUSG00000001627	Ifrd1	-0,705	-0,271	-0,715	0,002	0,003	0,002
ENSMUSG00000001663	Gstt1	3,501	1,666	1,284	0,001	0,000	0,172
ENSMUSG00000002342	Tmem161a	0,634	0,187	0,573	0,007	0,010	0,015
ENSMUSG00000003032	Klf4	0,927	0,271	0,833	0,006	0,003	0,016
ENSMUSG00000003528	Slc25a1	0,562	0,214	0,524	0,006	0,007	0,010
ENSMUSG00000003604	Aven	-0,613	-0,288	-0,572	0,010	0,002	0,015
ENSMUSG00000003617	Cp	2,547	0,053	2,451	0,003	0,083	0,009
ENSMUSG00000003721	Insig2	-0,979	-0,628	-0,912	0,002	0,000	0,004
ENSMUSG00000003779	Kif20a	1,012	0,191	0,898	0,009	0,008	0,020
ENSMUSG00000003992	Ssbp2	0,813	0,150	0,643	0,009	0,025	0,032
ENSMUSG00000004263	Atn1	-0,527	-0,309	-0,523	0,001	0,001	0,001
ENSMUSG00000005338	Cadm3	2,463	1,013	2,323	0,000	0,000	0,001
ENSMUSG00000005371	Fbxo11	-0,521	-0,367	-0,520	0,001	0,000	0,001
ENSMUSG00000005483	Dnajb1	-0,978	-0,346	-0,856	0,003	0,001	0,005
ENSMUSG00000005667	Mthfd2	1,918	0,412	1,688	0,003	0,001	0,012
ENSMUSG00000006310	Zbtb32	1,594	0,379	1,602	0,001	0,001	0,001
ENSMUSG00000006356	Crip2	1,280	0,736	1,285	0,000	0,000	0,000
ENSMUSG00000006705	Pknox1	-0,546	-0,283	-0,545	0,002	0,002	0,002
ENSMUSG00000007617	Homer1	-0,616	-0,371	-0,610	0,005	0,001	0,006
ENSMUSG00000012443	Kif11	0,900	0,201	0,945	0,009	0,008	0,005
ENSMUSG00000013833	Med16	0,689	0,383	0,688	0,001	0,000	0,001
ENSMUSG00000015127	Unkl	0,946	0,275	0,907	0,002	0,003	0,003
ENSMUSG00000015189	Casd1	-0,715	-0,222	-0,694	0,003	0,006	0,004
ENSMUSG00000015340	Cybb	0,969	0,223	0,961	0,008	0,005	0,012
ENSMUSG00000015568	Lpl	1,034	0,212	0,976	0,004	0,006	0,008
ENSMUSG00000015656	Hspa8	-0,793	-0,309	-0,777	0,010	0,002	0,007
ENSMUSG00000017607	Tns4	1,821	1,943	1,933	0,000	0,000	0,000
ENSMUSG00000018196	Glrx2	0,778	0,462	0,750	0,001	0,000	0,002
ENSMUSG00000018363	Smurf2	-0,491	-0,218	-0,476	0,008	0,006	0,008
ENSMUSG00000020038	Cry1	-0,472	-0,197	-0,452	0,008	0,011	0,011
ENSMUSG00000020085	Aifm2	1,537	0,133	1,281	0,010	0,016	0,047
ENSMUSG00000020102	Slc16a7	0,999	0,427	0,992	0,002	0,001	0,002
ENSMUSG00000020279	Il9r	1,298	0,348	1,274	0,002	0,001	0,003
ENSMUSG00000020287	Mpg	0,643	0,345	0,632	0,001	0,000	0,001
ENSMUSG00000020334	Slc22a4	1,383	0,224	1,539	0,005	0,004	0,002
ENSMUSG00000020437	Myo1g	0,504	0,248	0,518	0,007	0,003	0,005
ENSMUSG00000020482	Ccdc117	-0,569	-0,218	-0,525	0,009	0,007	0,012
ENSMUSG00000020547	Bzw2	-0,461	-0,281	-0,453	0,003	0,001	0,003
ENSMUSG00000020834	Dhrs13	-1,954	-0,999	-1,637	0,001	0,000	0,004
ENSMUSG00000020897	Aurkb	1,086	0,576	1,107	0,001	0,000	0,000
ENSMUSG00000020914	Top2a	0,786	0,123	0,795	0,006	0,050	0,005
ENSMUSG00000021067	Sav1	-0,568	-0,236	-0,525	0,005	0,005	0,007
ENSMUSG00000021103	Mnat1	0,681	0,204	0,674	0,006	0,009	0,006
ENSMUSG00000021156	Zmynd11	-0,590	-0,204	-0,610	0,004	0,008	0,003
ENSMUSG00000021495	Fam193b	-0,488	-0,372	-0,497	0,002	0,000	0,001
ENSMUSG00000021596	Mctp1	-1,080	-0,292	-1,044	0,006	0,002	0,004
ENSMUSG00000021624	Cd180	0,803	0,462	0,847	0,001	0,000	0,001
ENSMUSG00000021879	Dnah12	1,993	3,209	0,859	0,005	0,000	0,218
ENSMUSG00000021967	Mrpl57	0,497	0,406	0,490	0,004	0,000	0,004
ENSMUSG00000022105	Rb1	0,652	0,195	0,629	0,007	0,011	0,011
ENSMUSG00000022440	C1qtnf6	1,173	0,430	1,197	0,001	0,001	0,000

ENSMUSG00000022462	Slc38a2	-0,559	-0,290	-0,561	0,003	0,001	0,002
ENSMUSG00000022586	Ly6i	1,182	0,210	1,150	0,009	0,006	0,018
ENSMUSG00000022601	Zbtb11	-0,587	-0,352	-0,598	0,003	0,000	0,003
ENSMUSG00000022742	Cpox	1,298	0,216	1,091	0,006	0,005	0,022
ENSMUSG00000022895	Ets2	-1,480	-1,073	-1,320	0,000	0,000	0,001
ENSMUSG00000023827	Agpat4	0,848	0,242	0,826	0,007	0,004	0,012
ENSMUSG00000024042	Sik1	-0,819	-0,232	-0,725	0,010	0,005	0,022
ENSMUSG00000024097	Srsf7	-0,963	-0,738	-0,950	0,000	0,000	0,000
ENSMUSG00000024137	E4f1	-0,413	-0,226	-0,417	0,007	0,006	0,005
ENSMUSG00000024222	Fkbp5	-1,407	-0,274	-1,160	0,010	0,003	0,021
ENSMUSG00000024330	Col11a2	-1,001	-0,356	-1,027	0,001	0,001	0,001
ENSMUSG00000024424	Ttc39c	1,264	0,252	1,313	0,004	0,003	0,002
ENSMUSG00000024431	Nr3c1	-0,540	-0,411	-0,526	0,001	0,000	0,001
ENSMUSG00000024597	Slc12a2	0,951	0,352	0,874	0,002	0,001	0,007
ENSMUSG00000024824	Rad9a	-0,446	-0,275	-0,445	0,009	0,001	0,007
ENSMUSG00000024966	Stip1	-1,297	-0,961	-1,186	0,000	0,000	0,000
ENSMUSG00000024982	Zdhhc6	0,637	0,334	0,635	0,003	0,001	0,003
ENSMUSG00000024983	Vti1a	0,390	0,132	0,379	0,006	0,045	0,006
ENSMUSG00000025140	Pycr1	2,594	0,660	1,958	0,001	0,001	0,020
ENSMUSG00000025316	Banp	-0,764	-0,323	-0,772	0,000	0,001	0,000
ENSMUSG00000025403	Shmt2	0,989	0,182	0,725	0,006	0,012	0,042
ENSMUSG00000025574	Tk1	0,846	0,174	0,814	0,003	0,014	0,004
ENSMUSG00000025810	Nrp1	1,319	0,828	1,364	0,000	0,000	0,000
ENSMUSG00000025958	Creb1	-0,478	-0,212	-0,472	0,003	0,007	0,003
ENSMUSG00000026014	Raph1	0,805	0,244	0,759	0,004	0,004	0,008
ENSMUSG00000026142	Rhbdd1	0,488	0,299	0,463	0,003	0,000	0,003
ENSMUSG00000026280	Atg4b	0,623	0,297	0,597	0,002	0,002	0,003
ENSMUSG00000026285	Pdcd1	2,071	1,627	1,794	0,000	0,000	0,001
ENSMUSG00000026307	Scly	0,618	0,235	0,609	0,003	0,005	0,004
ENSMUSG00000026765	Lypd6b	-0,792	-0,323	-0,785	0,003	0,001	0,004
ENSMUSG00000027132	Katnbl1	-0,746	-0,349	-0,722	0,001	0,001	0,002
ENSMUSG00000027358	Bmp2	-2,267	-0,487	-2,055	0,004	0,001	0,007
ENSMUSG00000027359	Slc27a2	-1,588	-0,177	-1,387	0,009	0,008	0,027
ENSMUSG00000027387	Zc3h8	-0,820	-0,280	-0,812	0,005	0,002	0,007
ENSMUSG00000027401	Tgm3	1,169	0,263	1,068	0,004	0,003	0,009
ENSMUSG00000027858	Tspan2	-1,091	-0,355	-1,094	0,003	0,001	0,002
ENSMUSG00000028037	Ifi44	2,240	0,780	2,086	0,001	0,000	0,002
ENSMUSG00000028173	Wls	0,729	0,569	0,718	0,000	0,000	0,000
ENSMUSG00000028217	Cdh17	1,499	0,234	1,602	0,006	0,004	0,005
ENSMUSG00000028410	Dnaja1	-1,434	-1,073	-1,311	0,000	0,000	0,000
ENSMUSG00000028412	Slc44a1	-0,888	-0,205	-0,849	0,009	0,007	0,008
ENSMUSG00000028476	Reck	0,962	0,556	0,911	0,004	0,000	0,005
ENSMUSG00000028518	Prkaa2	-1,780	-0,734	-1,612	0,002	0,000	0,004
ENSMUSG00000028551	Cdkn2c	0,983	0,164	0,991	0,010	0,013	0,009
ENSMUSG00000028776	Tinagl1	1,250	0,207	1,205	0,009	0,006	0,012
ENSMUSG00000028885	Smpdl3b	1,317	0,256	1,186	0,004	0,003	0,017
ENSMUSG00000029090	Adgra3	-2,595	-2,236	-2,372	0,001	0,000	0,003
ENSMUSG00000029504	Ddx51	-1,408	-2,551	-1,365	0,000	0,000	0,000
ENSMUSG00000029561	Oasl2	1,741	0,297	1,880	0,007	0,002	0,008
ENSMUSG00000029598	Plbd2	0,764	0,217	0,700	0,007	0,006	0,013
ENSMUSG00000029647	Pan3	-0,429	-0,174	-0,425	0,006	0,018	0,004
ENSMUSG00000029657	Hsph1	-1,875	-0,788	-1,570	0,001	0,000	0,002
ENSMUSG00000029720	Gm20605	-0,572	-0,340	-0,562	0,003	0,000	0,003
ENSMUSG00000029817	Tra2a	-0,747	-0,618	-0,747	0,000	0,000	0,000
ENSMUSG00000029923	Rab19	0,656	0,325	0,633	0,003	0,001	0,003
ENSMUSG00000030016	Zfp638	-0,528	-0,154	-0,543	0,009	0,024	0,006
ENSMUSG00000030103	Bhlhe40	0,808	0,439	0,817	0,001	0,000	0,001
ENSMUSG00000030124	Lag3	1,795	1,517	1,720	0,000	0,000	0,000
ENSMUSG00000030287	Itpr2	-0,953	-0,225	-0,884	0,008	0,005	0,010

ENSMUSG00000030357	Fkbp4	-0,970	-0,605	-0,939	0,001	0,000	0,000
ENSMUSG00000030738	Eif3c	0,863	0,327	0,736	0,002	0,001	0,008
ENSMUSG00000030792	Dkkl1	1,307	0,082	1,318	0,009	0,065	0,006
ENSMUSG00000030979	Uros	1,171	0,273	1,053	0,004	0,003	0,010
ENSMUSG00000031242	2610002M06F	0,988	0,804	0,957	0,000	0,000	0,001
ENSMUSG00000031349	Nsdhl	0,591	0,329	0,557	0,002	0,000	0,003
ENSMUSG00000031373	Car5b	0,919	0,283	0,889	0,005	0,002	0,004
ENSMUSG00000031431	Tsc22d3	-1,034	-0,685	-1,082	0,001	0,000	0,000
ENSMUSG00000031820	Babam1	0,368	0,205	0,364	0,009	0,005	0,009
ENSMUSG00000031960	Aars	0,761	0,065	0,762	0,006	0,177	0,006
ENSMUSG00000032026	Rexo2	1,333	0,556	1,057	0,001	0,000	0,005
ENSMUSG00000032218	Ccnb2	0,998	0,183	0,988	0,007	0,010	0,007
ENSMUSG00000032238	Rora	1,232	0,863	1,074	0,001	0,000	0,002
ENSMUSG00000032336	Nptn	-0,890	-0,240	-0,908	0,009	0,004	0,005
ENSMUSG00000032352	Lrrc1	-0,941	-0,290	-0,953	0,007	0,002	0,009
ENSMUSG00000032496	Ltf	4,419	3,355	4,304	0,000	0,000	0,001
ENSMUSG00000032507	Fbxl2	-1,012	-0,182	-0,979	0,009	0,010	0,009
ENSMUSG00000032515	Csrnp1	-0,751	-0,264	-0,702	0,004	0,003	0,005
ENSMUSG00000032715	Trib3	1,505	0,169	1,270	0,006	0,009	0,019
ENSMUSG00000032737	Inppl1	0,921	0,375	0,878	0,001	0,001	0,002
ENSMUSG00000032867	Fbxw8	0,699	0,348	0,710	0,002	0,001	0,001
ENSMUSG00000032915	Adgre4	0,940	0,214	0,937	0,008	0,006	0,011
ENSMUSG00000033111	3830406C13R	0,678	0,653	0,688	0,000	0,000	0,000
ENSMUSG00000033287	Kctd17	0,990	0,270	0,930	0,006	0,003	0,009
ENSMUSG00000033545	Znrf1	-0,535	-0,188	-0,521	0,009	0,013	0,009
ENSMUSG00000033918	Parl	0,559	0,242	0,529	0,005	0,004	0,009
ENSMUSG00000033960	Jcad	-1,505	-0,476	-1,351	0,003	0,001	0,009
ENSMUSG00000034120	Srsf2	-0,495	-0,200	-0,491	0,003	0,010	0,002
ENSMUSG00000034177	Rnf43	1,441	1,505	1,199	0,002	0,000	0,016
ENSMUSG00000034189	Hsdl1	-0,386	-0,259	-0,377	0,010	0,001	0,008
ENSMUSG00000034543	Morc2a	0,776	1,508	0,752	0,000	0,000	0,000
ENSMUSG00000034573	Ptpn13	1,470	0,262	1,274	0,008	0,003	0,026
ENSMUSG00000034620	Tmem5	0,609	0,310	0,587	0,007	0,002	0,008
ENSMUSG00000034730	Adgrb1	3,456	3,923	3,150	0,000	0,000	0,000
ENSMUSG00000034792	Gna15	0,765	0,217	0,703	0,005	0,006	0,008
ENSMUSG00000035042	Ccl5	1,399	1,106	1,321	0,000	0,000	0,000
ENSMUSG00000035504	Reep6	2,138	0,929	1,689	0,001	0,000	0,006
ENSMUSG00000035692	Isg15	1,337	0,217	1,297	0,006	0,005	0,012
ENSMUSG00000035851	Ythdc1	-0,520	-0,389	-0,529	0,001	0,000	0,001
ENSMUSG00000036206	Sh3bp4	1,108	0,175	1,018	0,009	0,010	0,022
ENSMUSG00000036304	Zdhhc23	-0,771	-0,385	-0,737	0,004	0,001	0,007
ENSMUSG00000036353	P2ry12	-1,192	-0,313	-1,118	0,005	0,002	0,004
ENSMUSG00000036395	Glb1l2	-0,873	-0,157	-0,829	0,010	0,019	0,012
ENSMUSG00000036779	Papd5	-0,805	-0,224	-0,811	0,006	0,006	0,005
ENSMUSG00000036931	Nfkbid	-0,452	-0,304	-0,443	0,003	0,000	0,003
ENSMUSG00000037089	Slc35b2	0,519	0,179	0,507	0,006	0,016	0,007
ENSMUSG00000037151	Lrrc20	0,824	0,323	0,840	0,004	0,001	0,002
ENSMUSG00000037572	Wdhd1	-1,117	-1,033	-0,982	0,003	0,000	0,014
ENSMUSG00000037573	Tob1	-0,598	-0,211	-0,576	0,009	0,008	0,012
ENSMUSG00000037816	Fbxw17	1,122	0,137	0,975	0,003	0,018	0,009
ENSMUSG00000038068	Rnf144b	-0,854	-0,236	-0,833	0,009	0,004	0,009
ENSMUSG00000038070	Cntln	1,423	0,562	1,630	0,002	0,001	0,002
ENSMUSG00000038126	Mphosph9	-1,089	-1,323	-1,067	0,005	0,000	0,007
ENSMUSG00000038150	Ormdl3	-0,955	-0,232	-0,908	0,010	0,005	0,009
ENSMUSG00000038357	Camp	4,746	2,998	4,349	0,000	0,000	0,001
ENSMUSG00000038417	Fig4	0,485	0,227	0,442	0,007	0,004	0,013
ENSMUSG00000038539	Atf5	3,545	2,064	2,502	0,001	0,000	0,019
ENSMUSG00000038943	Prc1	1,138	0,139	1,278	0,007	0,018	0,002
ENSMUSG00000039450	Dcxr	1,191	0,402	1,220	0,002	0,001	0,002

ENSMUSG00000039452	Snx22	1,040	0,193	1,009	0,008	0,008	0,009
ENSMUSG00000039480	Nt5dc1	0,387	0,302	0,385	0,006	0,000	0,005
ENSMUSG00000039662	Icmt	-0,361	-0,271	-0,361	0,010	0,000	0,008
ENSMUSG00000039787	Cercam	0,985	0,105	0,926	0,007	0,046	0,012
ENSMUSG00000039831	Arhgap29	-1,672	-0,346	-1,773	0,009	0,002	0,017
ENSMUSG00000039873	Neurl2	1,145	0,509	1,212	0,002	0,000	0,001
ENSMUSG00000039911	Spsb1	-0,965	-0,039	-0,947	0,009	0,369	0,014
ENSMUSG00000040018	Cox15	0,669	0,711	0,606	0,003	0,000	0,007
ENSMUSG00000040128	Pnrc1	-0,379	-0,175	-0,377	0,009	0,013	0,006
ENSMUSG00000040167	Ikzf5	0,553	0,168	0,532	0,008	0,020	0,010
ENSMUSG00000040253	Gbp7	0,847	0,247	0,844	0,005	0,004	0,008
ENSMUSG00000040415	Dtx3	0,677	0,520	0,666	0,000	0,000	0,000
ENSMUSG00000040738	Ints8	-0,687	-0,273	-0,680	0,003	0,003	0,003
ENSMUSG00000040811	Eml2	0,599	0,207	0,582	0,007	0,009	0,007
ENSMUSG00000041073	Nacad	2,527	0,494	2,579	0,001	0,001	0,002
ENSMUSG00000041390	Mdfic	0,556	0,255	0,533	0,005	0,003	0,006
ENSMUSG00000041431	Ccnb1	1,101	0,069	0,986	0,005	0,122	0,013
ENSMUSG00000041481	Serpina3g	0,939	0,317	0,859	0,002	0,001	0,005
ENSMUSG00000041483	Zfp281	-0,558	-0,298	-0,542	0,002	0,001	0,002
ENSMUSG00000041491	Cep78	0,655	0,262	0,630	0,007	0,003	0,008
ENSMUSG00000041633	Kctd12b	0,974	0,302	0,941	0,003	0,002	0,006
ENSMUSG00000042029	Ncapg2	0,951	0,270	0,939	0,003	0,003	0,003
ENSMUSG00000042032	Mat2b	0,910	0,200	0,844	0,008	0,008	0,016
ENSMUSG00000042385	Gzmk	2,245	1,296	2,300	0,000	0,000	0,000
ENSMUSG00000042726	Trafd1	0,884	0,199	0,924	0,007	0,008	0,007
ENSMUSG00000043243	Fam129c	-1,464	-0,265	-1,517	0,006	0,003	0,015
ENSMUSG00000043505	Gimap5	0,443	0,562	0,449	0,006	0,000	0,006
ENSMUSG00000043929	Klhl15	0,761	0,256	0,667	0,006	0,003	0,017
ENSMUSG00000043932	Klri2	-0,845	-0,214	-0,839	0,009	0,007	0,011
ENSMUSG00000044252	Osbpl1a	1,861	1,316	1,847	0,000	0,000	0,000
ENSMUSG00000044636	Csrnp2	-0,884	-0,309	-0,882	0,004	0,002	0,004
ENSMUSG00000044645	Gm7334	0,945	0,165	0,903	0,010	0,014	0,018
ENSMUSG00000044703	Phf11a	0,971	0,246	1,026	0,006	0,004	0,005
ENSMUSG00000045106	Ccdc73	1,088	0,202	1,186	0,007	0,006	0,004
ENSMUSG00000045328	Cenpe	0,777	0,072	0,806	0,009	0,098	0,005
ENSMUSG00000045382	Cxcr4	-0,976	-0,335	-1,027	0,005	0,001	0,003
ENSMUSG00000045691	Thtpa	0,631	0,210	0,623	0,009	0,008	0,010
ENSMUSG00000045868	Gvin1	2,396	2,154	2,311	0,000	0,000	0,000
ENSMUSG00000046442	Ppm1e	2,121	1,176	1,389	0,004	0,000	0,068
ENSMUSG00000047507	Baiap3	0,910	0,170	0,866	0,005	0,013	0,010
ENSMUSG00000047875	Gpr157	1,360	0,453	1,569	0,004	0,001	0,002
ENSMUSG00000048058	Ldlrad3	1,075	0,262	1,012	0,004	0,003	0,010
ENSMUSG00000048249	Crebrf	-0,508	-0,224	-0,485	0,007	0,006	0,008
ENSMUSG00000048644	Ctxn1	-0,975	-0,295	-0,900	0,005	0,002	0,005
ENSMUSG00000049119	Fam110b	-1,235	-0,250	-1,102	0,006	0,003	0,015
ENSMUSG00000049608	Gpr55	0,875	0,387	0,857	0,001	0,001	0,001
ENSMUSG00000050014	Apol10b	2,664	1,828	2,952	0,000	0,000	0,000
ENSMUSG00000050232	Cxcr3	1,250	0,788	1,103	0,000	0,000	0,001
ENSMUSG00000050244	Heatr1	-0,495	-0,560	-0,497	0,010	0,000	0,009
ENSMUSG00000050410	Tcf19	0,739	0,167	0,744	0,008	0,017	0,008
ENSMUSG00000050912	Tmem123	-0,741	-0,245	-0,731	0,006	0,004	0,005
ENSMUSG00000051457	Spn	0,633	0,252	0,676	0,004	0,003	0,003
ENSMUSG00000051730	Mettl5	0,675	0,255	0,617	0,010	0,004	0,016
ENSMUSG00000052336	Cx3cr1	1,291	0,691	1,343	0,001	0,000	0,001
ENSMUSG00000053080	2700081O15R	-1,835	-0,962	-1,762	0,003	0,000	0,009
ENSMUSG00000053907	Mat2a	-0,756	-0,259	-0,718	0,008	0,003	0,009
ENSMUSG00000055963	Triqk	-1,712	-0,237	-1,390	0,009	0,004	0,031
ENSMUSG00000056116	H2-T22	0,665	0,527	0,652	0,000	0,000	0,000
ENSMUSG00000056737	Capg	0,654	0,187	0,645	0,009	0,013	0,013

ENSMUSG00000057367	Birc2	-0,912	-0,258	-0,919	0,004	0,003	0,003
ENSMUSG00000057409	Zfp53	-0,633	-0,406	-0,612	0,001	0,000	0,002
ENSMUSG00000057858	Fam204a	0,531	0,369	0,526	0,004	0,000	0,004
ENSMUSG00000058183	Mmel1	1,817	0,176	1,629	0,003	0,008	0,009
ENSMUSG00000058216	Gstp3	1,374	0,915	1,328	0,000	0,000	0,000
ENSMUSG00000059336	Slc14a1	0,803	0,171	0,700	0,004	0,014	0,011
ENSMUSG00000059674	Cdh24	-1,437	-0,321	-1,421	0,001	0,002	0,001
ENSMUSG00000060002	Chpt1	0,947	0,704	0,907	0,002	0,000	0,002
ENSMUSG00000060600	Eno3	1,101	0,291	1,086	0,001	0,002	0,002
ENSMUSG00000060798	Intu	-1,266	-0,378	-1,188	0,002	0,001	0,005
ENSMUSG00000060803	Gstp1	0,581	0,218	0,548	0,005	0,007	0,007
ENSMUSG00000062175	Tgif2	-0,614	-0,322	-0,640	0,009	0,001	0,008
ENSMUSG00000062376	Borcs7	0,635	0,303	0,623	0,007	0,001	0,006
ENSMUSG00000063235	Ptpmt1	0,818	0,371	0,811	0,002	0,001	0,003
ENSMUSG00000063245	Zfp993	2,160	1,719	2,248	0,004	0,000	0,004
ENSMUSG00000063972	Nr6a1	-1,163	-0,106	-1,175	0,007	0,038	0,008
ENSMUSG00000064090	Vrk2	0,710	0,290	0,688	0,002	0,002	0,002
ENSMUSG00000067220	Cnga1	-1,545	-0,418	-1,599	0,003	0,001	0,007
ENSMUSG00000067847	Romo1	0,614	0,308	0,668	0,004	0,001	0,001
ENSMUSG00000068220	Lgals1	0,817	0,202	0,832	0,008	0,008	0,008
ENSMUSG00000068606	Gm4841	2,143	1,727	2,111	0,000	0,000	0,000
ENSMUSG00000068739	Sars	0,598	0,338	0,586	0,001	0,000	0,001
ENSMUSG00000068749	Psma5	0,619	0,210	0,554	0,008	0,008	0,017
ENSMUSG00000068758	Il3ra	0,780	0,385	0,724	0,007	0,001	0,012
ENSMUSG00000068917	Clk2	-0,515	-0,305	-0,519	0,007	0,001	0,006
ENSMUSG00000071072	Ptges3	-0,750	-0,380	-0,724	0,001	0,000	0,001
ENSMUSG00000071172	Srsf3	-0,468	-0,301	-0,441	0,010	0,000	0,010
ENSMUSG00000071711	Mpst	1,541	0,228	1,205	0,007	0,004	0,036
ENSMUSG00000072082	Ccnf	0,933	0,321	0,940	0,003	0,001	0,001
ENSMUSG00000072596	Ear2	1,142	0,489	1,081	0,001	0,000	0,003
ENSMUSG00000072647	Adam1a	-0,940	-0,431	-0,946	0,001	0,000	0,001
ENSMUSG00000072770	Acrbp	-0,633	-0,174	-0,584	0,006	0,017	0,011
ENSMUSG00000072872	Rybp	-0,517	-0,270	-0,499	0,003	0,002	0,003
ENSMUSG00000073542	Cep76	0,928	0,184	0,925	0,007	0,010	0,008
ENSMUSG00000074203	G430095P16R	0,975	0,363	0,990	0,004	0,001	0,002
ENSMUSG00000075327	Zbtb2	-0,522	-0,357	-0,510	0,002	0,000	0,002
ENSMUSG00000076612	Ighg2c	2,264	1,749	2,376	0,000	0,000	0,000
ENSMUSG00000076615	Ighg3	1,546	0,240	1,597	0,001	0,004	0,000
ENSMUSG00000078606	Gm4070	2,651	2,509	2,538	0,000	0,000	0,000
ENSMUSG00000079003	Samd1	-0,837	-0,666	-0,813	0,000	0,000	0,000
ENSMUSG00000079334	Nat6	0,552	0,348	0,532	0,005	0,001	0,007
ENSMUSG00000079442	St6galnac4	0,456	0,233	0,445	0,009	0,004	0,008
ENSMUSG00000081189	Hspd1-ps4	-1,287	-0,293	-1,187	0,005	0,002	0,009
ENSMUSG00000081723	Gm15931	1,569	0,556	1,558	0,001	0,000	0,002
ENSMUSG00000085331	Gm11274	-1,454	-0,284	-1,430	0,006	0,002	0,011
ENSMUSG00000085622	3110056K07Ri	-0,613	-0,300	-0,609	0,002	0,001	0,002
ENSMUSG00000086438	Asb17os	2,061	0,642	1,902	0,001	0,000	0,004
ENSMUSG00000091650	Apol11a	3,003	2,366	3,141	0,000	0,000	0,000
ENSMUSG00000091694	Apol11b	2,549	2,138	2,586	0,000	0,000	0,000
ENSMUSG00000092060	Bend4	-1,132	-0,581	-1,056	0,001	0,000	0,001
ENSMUSG00000095079	Igha	-2,014	-0,687	-2,127	0,005	0,000	0,006
ENSMUSG00000095217	Hist1h2bn	-1,201	-0,283	-1,105	0,004	0,002	0,008
ENSMUSG00000096210	H1f0	1,115	0,403	1,094	0,002	0,001	0,003
ENSMUSG00000097838	C530050E15Ri	-1,434	-0,335	-1,377	0,005	0,002	0,005
ENSMUSG00000098912	1500004A13R	0,910	0,157	0,822	0,009	0,013	0,015
ENSMUSG00000098950	Gm28036	-1,285	-0,457	-1,429	0,008	0,001	0,008
ENSMUSG00000099474	1700097N02R	1,755	1,195	1,581	0,000	0,000	0,001
ENSMUSG00000099974	Bcl2a1d	1,043	0,372	1,025	0,001	0,001	0,003
ENSMUSG00000106219	5830416I19Ril	-1,112	-0,398	-1,052	0,002	0,001	0,006

ENSMUSG00000106438	Gm32051	3,471	2,855	2,485	0,000	0,000	0,002
ENSMUSG00000107331	Gm42732	-1,589	-0,404	-1,446	0,003	0,001	0,005
ENSMUSG00000108218	Olfr1372-ps1	-0,870	-0,323	-0,757	0,004	0,001	0,010
ENSMUSG00000109675	Nxpe1-ps	0,875	0,220	0,879	0,006	0,006	0,006
ENSMUSG00000109704	Gm38414	-1,688	-0,946	-1,570	0,001	0,000	0,001
ENSMUSG00000109941	Exosc6	-8,439	-0,114	-4,520	0,005	0,001	0,029
ENSMUSG00000112662	Gm47922	-0,902	-0,376	-0,966	0,004	0,001	0,002
ENSMUSG00000113136	Gm19951	-0,970	-0,564	-0,981	0,001	0,000	0,001
ENSMUSG00000114635	Gm49392	-2,069	-0,610	-2,152	0,005	0,001	0,008
ENSMUSG00000002221	Paxip1	-0,500	-0,247	-0,518	0,057	0,004	0,056
ENSMUSG00000002343	Armc6	-0,836	-0,222	-0,749	0,019	0,005	0,039
ENSMUSG00000002504	Slc9a3r2	-1,044	-0,195	-0,989	0,028	0,007	0,044
ENSMUSG00000002578	Ikzf4	1,104	2,048	1,063	0,019	0,000	0,029
ENSMUSG00000002635	Pdcd2l	0,408	0,211	0,389	0,096	0,008	0,108
ENSMUSG00000003948	Mmd	-1,042	-0,221	-1,038	0,011	0,005	0,008
ENSMUSG00000004317	Clcn5	0,677	0,253	0,577	0,040	0,003	0,073
ENSMUSG00000005417	Mprip	-0,309	-0,233	-0,307	0,064	0,004	0,054
ENSMUSG00000005621	Zfp592	-0,336	-0,218	-0,314	0,053	0,007	0,061
ENSMUSG00000006611	Hfe	1,163	0,206	1,108	0,014	0,006	0,026
ENSMUSG00000006932	Ctnnb1	-0,325	-0,226	-0,324	0,037	0,004	0,027
ENSMUSG00000010054	Tusc2	-0,398	-0,210	-0,379	0,016	0,006	0,019
ENSMUSG00000010277	2610507B11R	-0,302	-0,218	-0,322	0,108	0,006	0,074
ENSMUSG00000012819	Cdh23	-1,216	-0,255	-1,018	0,027	0,003	0,070
ENSMUSG00000014039	Prdm15	-0,542	-0,232	-0,566	0,028	0,005	0,027
ENSMUSG00000015291	Gdi1	-0,356	-0,230	-0,357	0,043	0,005	0,033
ENSMUSG00000015355	Cd48	0,454	0,217	0,473	0,012	0,005	0,010
ENSMUSG00000015961	Adss	0,449	0,267	0,467	0,012	0,001	0,010
ENSMUSG00000017286	Glod4	0,459	0,211	0,468	0,034	0,008	0,029
ENSMUSG00000018661	Cog1	-0,238	-0,233	-0,241	0,068	0,004	0,055
ENSMUSG00000019837	Gtf3c6	0,399	0,233	0,371	0,021	0,005	0,032
ENSMUSG00000020048	Hsp90b1	-0,416	-0,198	-0,414	0,019	0,009	0,014
ENSMUSG00000020267	Hint1	0,631	0,220	0,583	0,012	0,007	0,018
ENSMUSG00000020280	Pus10	0,462	0,225	0,434	0,014	0,005	0,018
ENSMUSG00000020288	Ahsa2	-0,817	-0,191	-0,745	0,014	0,008	0,020
ENSMUSG00000020640	Itsn2	-0,490	-0,269	-0,490	0,012	0,003	0,009
ENSMUSG00000020739	Nup85	-0,634	-0,341	-0,622	0,030	0,001	0,039
ENSMUSG00000021076	Actr10	0,391	0,202	0,366	0,037	0,010	0,047
ENSMUSG00000021113	Snapc1	-0,788	-0,183	-0,833	0,017	0,008	0,014
ENSMUSG00000021676	Iqgap2	0,639	0,202	0,634	0,011	0,010	0,013
ENSMUSG00000021703	Serinc5	-0,573	-0,220	-0,550	0,012	0,007	0,015
ENSMUSG00000021945	Zmym2	-0,428	-0,254	-0,421	0,033	0,004	0,032
ENSMUSG00000022000	Zc3h13	-0,566	-0,235	-0,611	0,010	0,004	0,005
ENSMUSG00000022142	Nup155	-0,428	-0,221	-0,450	0,048	0,006	0,043
ENSMUSG00000022160	Mettl3	-0,590	-0,225	-0,611	0,020	0,005	0,018
ENSMUSG00000022884	Eif4a2	-0,523	-0,210	-0,520	0,019	0,008	0,017
ENSMUSG00000022911	Arl13b	-0,677	-0,185	-0,701	0,026	0,010	0,026
ENSMUSG00000023015	Racgap1	0,496	0,203	0,501	0,010	0,009	0,009
ENSMUSG00000023072	Cep89	-1,033	-0,536	-0,859	0,023	0,001	0,075
ENSMUSG00000023938	Aars2	-0,513	-0,199	-0,511	0,043	0,010	0,049
ENSMUSG00000024948	Map4k2	-0,482	-0,210	-0,462	0,025	0,008	0,025
ENSMUSG00000025650	Col7a1	2,205	0,151	2,265	0,018	0,005	0,029
ENSMUSG00000025791	Pgm2	0,495	0,220	0,484	0,011	0,006	0,011
ENSMUSG00000025899	Alkbh8	-0,487	-0,220	-0,438	0,051	0,006	0,096
ENSMUSG00000025940	Tmem70	0,272	0,196	0,258	0,072	0,007	0,078
ENSMUSG00000026031	Cflar	-0,706	-0,180	-0,694	0,012	0,010	0,009
ENSMUSG00000026131	Dst	1,254	0,445	1,387	0,030	0,001	0,010
ENSMUSG00000026353	Ubxn4	-0,452	-0,210	-0,447	0,019	0,008	0,016
ENSMUSG00000026357	Rgs18	-1,009	-0,206	-1,015	0,010	0,006	0,006
ENSMUSG00000026471	Mr1	0,779	0,260	0,729	0,010	0,003	0,017

ENSMUSG00000026484	Rnf2	-0,385	-0,198	-0,408	0,025	0,009	0,016
ENSMUSG00000026605	Cenpf	-0,971	-0,160	0,075	0,252	0,005	0,925
ENSMUSG00000026617	Bpnt1	-0,349	-0,216	-0,355	0,060	0,007	0,051
ENSMUSG00000026705	Klhl20	-0,307	-0,206	-0,319	0,063	0,008	0,054
ENSMUSG00000026843	Fubp3	0,744	0,286	0,657	0,016	0,002	0,033
ENSMUSG00000026994	Galnt3	1,448	0,187	1,449	0,030	0,006	0,030
ENSMUSG00000027087	Itgav	0,621	0,182	0,583	0,045	0,009	0,068
ENSMUSG00000027428	Rbbp9	-0,579	-0,207	-0,539	0,020	0,009	0,033
ENSMUSG00000027522	Stx16	-0,259	-0,202	-0,258	0,045	0,007	0,036
ENSMUSG00000027630	Tbl1xr1	-0,404	-0,212	-0,384	0,038	0,008	0,037
ENSMUSG00000027651	Rprd1b	0,458	0,271	0,434	0,017	0,002	0,023
ENSMUSG00000028132	Tmem56	1,182	0,173	1,030	0,064	0,008	0,129
ENSMUSG00000028689	Ccdc163	0,654	0,260	0,674	0,011	0,003	0,008
ENSMUSG00000028771	Ptpn12	-0,725	-0,271	-0,762	0,012	0,003	0,006
ENSMUSG00000028980	H6pd	0,615	0,200	0,595	0,021	0,010	0,025
ENSMUSG00000029050	Ski	-0,446	-0,228	-0,447	0,014	0,004	0,012
ENSMUSG00000029068	Ccnl2	-0,391	-0,232	-0,375	0,020	0,003	0,019
ENSMUSG00000029201	Ugdh	0,339	0,206	0,349	0,018	0,005	0,014
ENSMUSG00000029535	Triap1	-0,638	-0,222	-0,564	0,011	0,006	0,016
ENSMUSG00000030342	Cd9	-0,970	-0,201	-0,936	0,017	0,007	0,013
ENSMUSG00000030659	Nucb2	0,829	0,238	0,818	0,011	0,004	0,010
ENSMUSG00000030934	Oat	-0,328	-0,215	-0,323	0,014	0,002	0,011
ENSMUSG00000031148	Gpkow	-0,314	-0,198	-0,301	0,069	0,010	0,076
ENSMUSG00000031153	Gripap1	-0,452	-0,229	-0,421	0,011	0,005	0,012
ENSMUSG00000031167	Rbm3	0,616	0,233	0,629	0,016	0,005	0,013
ENSMUSG00000031226	Pbdc1	0,303	0,206	0,299	0,060	0,008	0,060
ENSMUSG00000031398	Plxna3	-1,745	-0,234	-1,691	0,033	0,003	0,074
ENSMUSG00000031422	Morf4l2	-0,447	-0,253	-0,455	0,010	0,002	0,007
ENSMUSG00000031516	Dctn6	0,331	0,207	0,333	0,013	0,005	0,010
ENSMUSG00000031633	Slc25a4	0,436	0,225	0,440	0,011	0,004	0,010
ENSMUSG00000031668	Eif2ak3	-0,423	-0,253	-0,406	0,016	0,002	0,017
ENSMUSG00000031770	Herpud1	-0,306	-0,283	-0,309	0,017	0,000	0,010
ENSMUSG00000032010	Usp2	-1,358	-0,171	-0,785	0,070	0,007	0,323
ENSMUSG00000032175	Tyk2	0,447	0,215	0,457	0,015	0,005	0,015
ENSMUSG00000032536	Trak1	-0,285	-0,202	-0,274	0,062	0,008	0,062
ENSMUSG00000032549	Rab6b	-1,076	-0,236	-1,093	0,011	0,004	0,019
ENSMUSG00000032637	Atxn2l	-0,279	-0,181	-0,260	0,067	0,010	0,070
ENSMUSG00000033427	Upb1	1,054	0,221	1,113	0,031	0,004	0,033
ENSMUSG00000034667	Xpot	-0,693	-0,240	-0,672	0,013	0,004	0,020
ENSMUSG00000035270	Impg2	-1,547	-0,978	-1,058	0,016	0,000	0,094
ENSMUSG00000035623	Rsf1	-0,448	-0,239	-0,455	0,041	0,005	0,038
ENSMUSG00000035697	Arhgap45	-0,243	-0,197	-0,239	0,095	0,001	0,078
ENSMUSG00000035891	Cerk	-0,434	-0,211	-0,427	0,019	0,007	0,016
ENSMUSG00000036948	BC037034	-0,482	-0,204	-0,473	0,023	0,009	0,020
ENSMUSG00000037152	Ndufc1	0,500	0,211	0,532	0,034	0,008	0,024
ENSMUSG00000037270	4932438A13R	-0,630	-0,232	-0,674	0,031	0,005	0,023
ENSMUSG00000037296	Lsm1	-0,443	-0,192	-0,416	0,042	0,010	0,062
ENSMUSG00000037466	Tedc1	0,659	0,164	0,573	0,135	0,010	0,199
ENSMUSG00000037499	Nenf	0,848	0,195	0,928	0,013	0,009	0,007
ENSMUSG00000037710	Cisd1	0,510	0,206	0,475	0,019	0,009	0,025
ENSMUSG00000037795	N4bp2	-0,827	-0,187	-0,815	0,037	0,008	0,059
ENSMUSG00000038587	Akap12	-1,119	-0,189	-1,094	0,015	0,008	0,040
ENSMUSG00000038611	Phrf1	-0,435	-0,235	-0,461	0,035	0,005	0,025
ENSMUSG00000038615	Nfe2l1	-0,281	-0,235	-0,268	0,055	0,003	0,057
ENSMUSG00000038831	Ralgps1	-0,764	-0,211	-0,679	0,023	0,006	0,054
ENSMUSG00000039254	Pomt1	-0,534	-0,304	-0,519	0,012	0,002	0,015
ENSMUSG00000039294	Cybc1	-0,533	-0,265	-0,545	0,015	0,003	0,009
ENSMUSG00000039428	Tmem135	0,369	0,198	0,359	0,013	0,007	0,014
ENSMUSG00000039458	Mtmr12	-0,215	-0,186	-0,206	0,109	0,006	0,112

ENSMUSG00000039648	Kyat1	-0,624	-0,205	-0,600	0,027	0,008	0,039
ENSMUSG00000040189	Ccdc114	-0,882	-0,286	-0,878	0,026	0,002	0,034
ENSMUSG00000040746	Rnf167	-0,353	-0,297	-0,337	0,023	0,000	0,019
ENSMUSG00000040855	Reps2	0,761	0,383	0,504	0,104	0,001	0,257
ENSMUSG00000041459	Tardbp	-0,377	-0,212	-0,381	0,017	0,005	0,012
ENSMUSG00000041460	Cacna2d4	0,835	0,453	0,619	0,093	0,001	0,240
ENSMUSG00000041720	Pi4ka	-0,413	-0,280	-0,419	0,032	0,002	0,026
ENSMUSG00000041977	Arhgef11	-0,386	-0,217	-0,375	0,033	0,005	0,036
ENSMUSG00000042213	Zfand4	-1,007	-0,184	-1,004	0,029	0,008	0,028
ENSMUSG00000042215	Bag2	0,943	0,214	1,052	0,013	0,005	0,005
ENSMUSG00000045268	Zfp691	0,459	0,307	0,460	0,077	0,002	0,067
ENSMUSG00000045969	Ing1	-0,380	-0,224	-0,376	0,012	0,004	0,009
ENSMUSG00000045975	C2cd2	-0,794	-0,277	-0,856	0,014	0,003	0,010
ENSMUSG00000046080	Clec9a	-0,969	-0,200	-0,674	0,050	0,006	0,191
ENSMUSG00000047388	Atmin	0,373	0,194	0,360	0,014	0,007	0,016
ENSMUSG00000048000	Gigyf2	-0,430	-0,320	-0,426	0,012	0,001	0,011
ENSMUSG00000048174	Tmem81	-0,558	-0,249	-0,551	0,011	0,004	0,014
ENSMUSG00000048756	Foxo3	0,629	0,181	0,570	0,052	0,009	0,082
ENSMUSG00000049001	Ndnf	0,857	0,467	0,630	0,057	0,001	0,157
ENSMUSG00000049191	Rtl5	-1,211	-1,454	-1,171	0,013	0,000	0,018
ENSMUSG00000049658	Bdp1	-0,496	-0,277	-0,462	0,016	0,002	0,024
ENSMUSG00000050565	Tor1aip2	-0,303	-0,270	-0,293	0,026	0,001	0,025
ENSMUSG00000050786	Ccdc126	-1,066	-0,190	-0,865	0,012	0,008	0,041
ENSMUSG00000051339	2900026A02R	-0,920	-0,188	-0,867	0,013	0,009	0,023
ENSMUSG00000051403	Ppp1r37	-0,414	-0,215	-0,417	0,020	0,007	0,018
ENSMUSG00000052605	Isoc2b	0,981	0,300	1,038	0,011	0,002	0,008
ENSMUSG00000052794	1700030K09Ri	-0,896	-0,216	-0,885	0,019	0,006	0,029
ENSMUSG00000053110	Yap1	-1,011	-0,208	-0,992	0,012	0,006	0,013
ENSMUSG00000053819	Camk2d	0,395	0,249	0,387	0,012	0,001	0,013
ENSMUSG00000054509	Parp4	-0,411	-0,259	-0,416	0,057	0,003	0,052
ENSMUSG00000055053	Nfic	0,585	0,232	0,575	0,015	0,005	0,018
ENSMUSG00000055435	Maf	0,409	0,216	0,413	0,020	0,007	0,016
ENSMUSG00000055491	Pprc1	-0,587	-0,206	-0,552	0,030	0,008	0,045
ENSMUSG00000056531	Ccdc18	0,911	0,496	1,150	0,023	0,001	0,002
ENSMUSG00000058006	Mdn1	-0,886	-0,184	-1,161	0,087	0,007	0,043
ENSMUSG00000058587	Tmod3	-0,453	-0,196	-0,449	0,011	0,010	0,008
ENSMUSG00000058833	Rex1bd	0,589	0,212	0,548	0,011	0,008	0,016
ENSMUSG00000059456	Ptk2b	-0,171	-0,190	-0,175	0,192	0,003	0,154
ENSMUSG00000060519	Tor3a	0,406	0,230	0,411	0,014	0,004	0,011
ENSMUSG00000060678	Hist1h4c	-0,992	-0,198	-0,887	0,012	0,008	0,021
ENSMUSG00000061028	Clasrp	-0,511	-0,210	-0,497	0,015	0,008	0,019
ENSMUSG00000061079	Zfp143	-0,290	-0,237	-0,308	0,085	0,004	0,062
ENSMUSG00000061758	Akr1b10	0,367	0,198	0,363	0,017	0,008	0,017
ENSMUSG00000062585	Cnr2	-0,365	-0,201	-0,356	0,030	0,007	0,025
ENSMUSG00000066043	Phactr4	-0,322	-0,189	-0,328	0,042	0,010	0,034
ENSMUSG00000066441	Rdh11	-0,553	-0,217	-0,566	0,010	0,007	0,009
ENSMUSG00000066687	Zbtb16	-1,152	-0,185	-0,905	0,013	0,008	0,065
ENSMUSG00000067149	Jchain	-1,731	-0,257	-1,477	0,012	0,003	0,055
ENSMUSG00000067203	H2-K2	-0,501	-0,206	-0,528	0,016	0,009	0,012
ENSMUSG00000070868	Skint3	0,794	0,193	0,802	0,020	0,009	0,028
ENSMUSG00000073640	Rpl27-ps3	0,351	0,194	0,346	0,028	0,007	0,027
ENSMUSG00000074340	Ovgp1	-0,800	-0,211	-0,714	0,015	0,006	0,029
ENSMUSG00000074342	I830077J02Rik	-0,663	-0,193	-0,640	0,141	0,007	0,136
ENSMUSG00000074405	Zfp865	0,445	0,288	0,443	0,018	0,002	0,019
ENSMUSG00000074657	Kif5a	-1,383	-0,174	-1,101	0,063	0,006	0,163
ENSMUSG00000075023	Accsl	-1,452	-0,174	-1,510	0,109	0,006	0,123
ENSMUSG00000075266	Cenpw	0,585	0,288	0,531	0,011	0,002	0,016
ENSMUSG00000079694	Gm14862	0,977	0,192	1,053	0,011	0,008	0,004
ENSMUSG00000083282	Ctsf	1,171	0,173	0,910	0,010	0,010	0,042

ENSMUSG00000085436	Zfp335os	-0,582	-0,198	-0,607	0,026	0,010	0,018
ENSMUSG00000089940	Gm4117	0,352	0,252	0,211	0,266	0,003	0,487
ENSMUSG00000090336	Cfap97d2	-1,166	-0,169	-1,184	0,020	0,010	0,017
ENSMUSG00000090958	Lrrc32	0,591	0,196	0,566	0,072	0,008	0,086
ENSMUSG00000092035	Peg10	-1,316	-0,213	-1,289	0,013	0,005	0,013
ENSMUSG00000095325	Zfp870	0,746	0,203	0,708	0,027	0,006	0,035
ENSMUSG00000096929	A330023F24R	-1,467	-0,242	-1,198	0,012	0,004	0,048
ENSMUSG00000097141	Gm10524	0,383	0,215	0,377	0,016	0,005	0,016
ENSMUSG00000097352	C920009B18R	0,433	0,226	0,416	0,027	0,006	0,027
ENSMUSG00000097993	Ptprv	-0,923	-0,161	-1,010	0,105	0,008	0,108
ENSMUSG00000099689	Zfp383	-0,829	-0,212	-0,945	0,015	0,007	0,007
ENSMUSG00000102705	4632432E15Ri	-1,419	-0,240	-1,204	0,010	0,004	0,030
ENSMUSG00000103983	Gm20045	-0,597	-0,236	-0,646	0,012	0,005	0,007
ENSMUSG00000109179	Gm35339	-0,721	-0,243	-0,718	0,011	0,004	0,011
ENSMUSG00000112095	A130077B15R	0,340	0,201	0,331	0,016	0,005	0,016
ENSMUSG00000112496	Gm48764	-0,694	-0,220	-0,661	0,013	0,006	0,022
ENSMUSG00000112907	Gm47015	-0,398	-0,220	-0,406	0,014	0,004	0,011
ENSMUSG00000115801	AC160336.1	-0,816	-0,257	-0,847	0,010	0,003	0,006
ENSMUSG00000017390	Aldoc	0,686	0,086	0,754	0,017	0,123	0,009
ENSMUSG00000021025	Nfkbia	-0,382	-0,180	-0,388	0,012	0,014	0,007
ENSMUSG00000025747	Tyms	1,119	0,144	1,226	0,014	0,012	0,006
ENSMUSG00000027620	Rbm39	-0,343	-0,136	-0,339	0,012	0,047	0,008
ENSMUSG00000027714	Exosc9	0,437	0,150	0,459	0,012	0,034	0,009
ENSMUSG00000028175	Depdc1a	0,842	0,118	1,055	0,037	0,033	0,005
ENSMUSG00000028820	Sfpq	-0,395	-0,143	-0,395	0,012	0,039	0,008
ENSMUSG00000028873	Cdca8	0,903	0,165	0,937	0,012	0,015	0,009
ENSMUSG00000029414	Kntc1	1,026	0,125	1,067	0,013	0,017	0,008
ENSMUSG00000039990	Edrf1	-0,615	-0,172	-0,676	0,014	0,012	0,008
ENSMUSG00000040028	Elavl1	-0,398	-0,184	-0,394	0,010	0,014	0,008
ENSMUSG00000041741	Pde3a	-0,819	-0,138	-1,046	0,033	0,026	0,008
ENSMUSG00000056656	Apol8	1,216	0,041	1,528	0,029	0,242	0,006
ENSMUSG00000056938	Acbd4	0,597	0,070	0,647	0,015	0,172	0,007
ENSMUSG00000062028	Irgc1	1,116	0,120	1,470	0,029	0,028	0,009
ENSMUSG00000074476	Spc24	0,986	0,097	0,995	0,013	0,055	0,009
ENSMUSG00000074918	Inafm2	-0,725	-0,164	-0,787	0,021	0,019	0,010
ENSMUSG00000097254	C430042M11F	0,706	0,142	0,817	0,028	0,028	0,010

Table S2. Tumor all differential genes

ID	symbol	logFC_edger	logFC_deseq	logFC_voom	pvalue_edger	pvalue_deseq	pvalue_voom
ENSMUSG00000004655	Aqp1	-0,808	-0,388	-0,819	0,007	0,000	0,005
ENSMUSG00000006369	Fbln1	-1,219	-0,674	-1,208	0,003	0,000	0,002
ENSMUSG00000013974	Mcemp1	1,424	0,397	1,377	0,007	0,001	0,008
ENSMUSG00000015085	Entpd2	-0,822	-0,253	-0,831	0,007	0,006	0,007
ENSMUSG00000015647	Lama5	-1,430	-0,237	-1,484	0,006	0,006	0,005
ENSMUSG00000016206	H2-M3	0,933	0,247	0,927	0,009	0,007	0,010
ENSMUSG00000018927	Ccl6	1,787	0,594	1,777	0,005	0,000	0,005
ENSMUSG00000020717	Pecam1	-0,907	-0,396	-0,901	0,003	0,001	0,003
ENSMUSG00000020949	Fkbp3	1,155	0,261	1,096	0,010	0,005	0,013
ENSMUSG00000022102	Dok2	1,334	0,567	1,311	0,001	0,000	0,002
ENSMUSG00000022126	Acod1	1,800	1,241	1,804	0,002	0,000	0,001
ENSMUSG00000024395	Lims2	-2,297	-0,748	-2,112	0,004	0,000	0,018
ENSMUSG00000026463	Atp2b4	-0,838	-0,369	-0,828	0,010	0,001	0,009
ENSMUSG00000026483	Fam129a	-1,089	-0,339	-1,043	0,006	0,002	0,007
ENSMUSG00000026981	Il1rn	1,271	0,543	1,307	0,008	0,000	0,007
ENSMUSG00000027188	Pamr1	-1,169	-0,404	-1,158	0,005	0,001	0,006
ENSMUSG00000027805	Pfn2	0,703	0,296	0,704	0,008	0,002	0,006
ENSMUSG00000028600	Podn	-1,136	-0,443	-1,148	0,003	0,000	0,003
ENSMUSG00000028776	Tinagl1	-1,110	-0,266	-1,136	0,010	0,004	0,009
ENSMUSG00000028874	Fgr	1,179	0,268	1,112	0,009	0,004	0,015
ENSMUSG00000029082	Bst1	1,181	0,363	1,130	0,004	0,001	0,006
ENSMUSG00000031432	Prps1	0,855	0,349	0,867	0,004	0,001	0,004
ENSMUSG00000031444	F10	1,818	0,406	1,955	0,006	0,001	0,003
ENSMUSG00000034872	Gipc3	-2,574	-0,422	-2,404	0,005	0,000	0,013
ENSMUSG00000038587	Akap12	-1,809	-0,431	-1,669	0,008	0,001	0,017
ENSMUSG00000041577	Prelp	-1,369	-0,840	-1,338	0,001	0,000	0,001
ENSMUSG00000042284	Itga1	-1,495	-0,466	-1,430	0,004	0,000	0,007
ENSMUSG00000044645	Gm7334	-9,420	-8,175	-6,914	0,001	0,000	0,001
ENSMUSG00000046280	She	-1,236	-0,694	-1,228	0,005	0,000	0,004
ENSMUSG00000047878	A4galt	-1,190	-0,310	-1,172	0,010	0,002	0,013
ENSMUSG00000049103	Ccr2	1,427	0,612	1,467	0,004	0,000	0,003
ENSMUSG00000053687	Dpep2	1,293	0,397	1,260	0,008	0,001	0,009
ENSMUSG00000054641	Mmrn1	-2,149	-0,453	-2,077	0,008	0,000	0,016
ENSMUSG00000058927	Gm10053	1,073	0,286	1,070	0,008	0,003	0,008
ENSMUSG00000059412	Fxyd2	1,343	0,805	1,322	0,003	0,000	0,003
ENSMUSG00000060923	Acyp2	1,564	0,355	1,516	0,009	0,001	0,011
ENSMUSG00000062980	Cped1	-0,956	-0,413	-0,948	0,003	0,001	0,004
ENSMUSG00000063694	Cycs	1,101	0,365	1,085	0,006	0,001	0,005
ENSMUSG00000068606	Gm4841	2,939	2,561	2,947	0,000	0,000	0,000
ENSMUSG00000068614	Actc1	2,564	1,348	2,555	0,002	0,000	0,003
ENSMUSG00000071708	Sms	1,114	0,405	1,092	0,006	0,001	0,007
ENSMUSG00000073600	Prob1	2,130	0,346	2,150	0,009	0,001	0,009
ENSMUSG00000074677	Sirpb1c	2,563	0,524	2,810	0,008	0,000	0,007
ENSMUSG00000076545	Igkv4-72	-2,286	-0,470	-2,178	0,006	0,000	0,010
ENSMUSG00000079293	Clec7a	1,967	0,328	1,847	0,005	0,001	0,008
ENSMUSG00000079547	H2-DMb1	1,111	0,337	1,143	0,004	0,002	0,003
ENSMUSG00000089942	Pira2	1,064	0,325	1,065	0,008	0,002	0,007
ENSMUSG00000093385	A330044P14Ri	1,891	0,243	1,780	0,007	0,003	0,010
ENSMUSG00000095609	Gm21188	1,934	0,614	2,037	0,007	0,000	0,005
ENSMUSG00000096463	Gm21750	2,746	1,701	2,588	0,002	0,000	0,003
ENSMUSG00000100599	1700120C14Ri	2,795	0,394	2,908	0,010	0,000	0,007
ENSMUSG00000102037	Bcl2a1a	2,101	0,604	2,193	0,005	0,000	0,004
ENSMUSG00000107771	Gm8956	2,524	0,371	2,468	0,007	0,000	0,008
ENSMUSG00000112043	Gm48774	2,294	0,328	2,082	0,007	0,001	0,014
ENSMUSG00000112149	Gm47611	1,895	0,455	1,771	0,006	0,000	0,009
ENSMUSG00000112226	Gm48786	1,899	0,452	1,757	0,006	0,000	0,010
ENSMUSG00000000088	Cox5a	0,846	0,242	0,848	0,016	0,008	0,013
ENSMUSG00000001349	Cnn1	-2,640	-0,257	-2,584	0,025	0,001	0,033

ENSMUSG00000002908	Kcnn1	1,338	0,228	1,431	0,035	0,004	0,025
ENSMUSG00000002992	Apoc2	2,303	0,270	2,463	0,017	0,001	0,019
ENSMUSG00000003477	Inmt	-3,607	-0,500	-2,559	0,014	0,000	0,180
ENSMUSG00000003849	Nqo1	-1,950	-0,146	-1,619	0,044	0,009	0,091
ENSMUSG00000005583	Mef2c	2,374	0,237	2,426	0,023	0,002	0,017
ENSMUSG00000005611	Mrvi1	-2,124	-0,238	-1,722	0,023	0,002	0,074
ENSMUSG00000006281	Tep1	1,021	0,198	1,022	0,037	0,005	0,041
ENSMUSG00000010342	Tex14	1,610	0,153	1,805	0,070	0,003	0,055
ENSMUSG00000017309	Cd300lg	-0,885	-0,258	-0,890	0,028	0,005	0,026
ENSMUSG00000017314	Mpp2	-1,030	-0,276	-1,049	0,013	0,003	0,011
ENSMUSG00000017485	Top2b	-0,734	-0,380	-0,740	0,016	0,001	0,013
ENSMUSG00000018417	Myo1b	-1,108	-0,224	-1,082	0,017	0,007	0,021
ENSMUSG00000018599	Mief2	1,361	0,207	1,322	0,024	0,007	0,027
ENSMUSG00000018909	Arrb1	-0,703	-0,244	-0,701	0,018	0,007	0,018
ENSMUSG00000019122	Ccl9	2,018	0,213	1,986	0,023	0,003	0,030
ENSMUSG00000019132	BC005537	-0,301	-0,241	-0,299	0,195	0,009	0,157
ENSMUSG00000019320	Noxo1	-0,943	-0,220	-0,922	0,054	0,006	0,050
ENSMUSG00000019726	Lyst	-1,013	-0,170	-1,085	0,037	0,009	0,026
ENSMUSG00000019775	Rgs17	1,652	0,221	1,821	0,033	0,002	0,024
ENSMUSG00000019848	Popdc3	2,467	0,173	2,560	0,029	0,004	0,023
ENSMUSG00000019979	Apaf1	0,841	1,229	0,868	0,012	0,000	0,011
ENSMUSG00000020155	Kcnmb1	-3,216	-0,374	-2,974	0,014	0,000	0,048
ENSMUSG00000020173	Cobl	2,620	0,112	2,692	0,030	0,005	0,023
ENSMUSG00000020231	Dip2a	-0,766	-0,246	-0,792	0,029	0,006	0,024
ENSMUSG00000020297	Nsg2	-2,419	-0,190	-2,101	0,026	0,003	0,069
ENSMUSG00000020307	Cdc34	1,229	0,202	1,196	0,018	0,009	0,018
ENSMUSG00000020386	Sar1b	1,271	0,226	1,216	0,019	0,007	0,019
ENSMUSG00000020390	Ube2b	1,105	0,210	1,063	0,023	0,009	0,025
ENSMUSG00000020541	Tom1l1	-1,073	-0,246	-1,059	0,031	0,004	0,037
ENSMUSG00000020682	Mmp28	-1,704	-0,212	-1,660	0,021	0,005	0,029
ENSMUSG00000020787	P2rx1	-2,744	-0,127	-2,880	0,053	0,006	0,097
ENSMUSG00000020812	1810032O08Ri	1,777	0,163	1,207	0,053	0,005	0,235
ENSMUSG00000020865	Abcc3	-1,456	-0,223	-1,486	0,050	0,006	0,046
ENSMUSG00000020908	Myh3	2,102	0,281	2,319	0,011	0,001	0,005
ENSMUSG00000020990	Cdkl1	-2,523	-0,189	-2,054	0,025	0,002	0,097
ENSMUSG00000021379	Id4	-2,242	-0,304	-1,894	0,014	0,001	0,071
ENSMUSG00000021384	Susd3	1,267	0,297	1,177	0,014	0,002	0,024
ENSMUSG00000021451	Sema4d	1,292	0,164	1,120	0,041	0,009	0,065
ENSMUSG00000021536	Adcy2	1,882	0,160	1,618	0,031	0,009	0,057
ENSMUSG00000021611	Tert	-1,253	-0,203	-1,208	0,024	0,004	0,025
ENSMUSG00000021767	Kat6b	-0,781	-0,240	-0,760	0,014	0,007	0,017
ENSMUSG00000021835	Bmp4	-1,066	-0,244	-1,070	0,022	0,004	0,025
ENSMUSG00000022013	Dnajc15	1,496	0,430	1,403	0,010	0,001	0,015
ENSMUSG00000022018	Rgcc	1,475	0,185	1,336	0,024	0,009	0,050
ENSMUSG00000022032	Scara5	-2,570	-0,321	-2,033	0,015	0,001	0,071
ENSMUSG00000022197	Pdzd2	-1,692	-0,159	-1,806	0,043	0,008	0,034
ENSMUSG00000022223	Sdr39u1	0,659	0,237	0,627	0,033	0,009	0,039
ENSMUSG00000022534	Mefv	1,577	0,183	1,619	0,026	0,009	0,027
ENSMUSG00000022668	Gtpbp8	1,157	0,316	0,997	0,029	0,001	0,061
ENSMUSG00000022836	Mylk	-2,301	-0,149	-2,465	0,050	0,005	0,066
ENSMUSG00000023861	Mpc1	1,072	0,260	1,055	0,013	0,005	0,012
ENSMUSG00000023982	Guca1a	1,696	0,236	1,537	0,017	0,003	0,039
ENSMUSG00000024059	Clip4	1,764	0,210	1,636	0,020	0,005	0,028
ENSMUSG00000024228	Nudt12	-1,096	-0,295	-1,008	0,029	0,002	0,055
ENSMUSG00000024483	Ankhd1	-0,874	-0,225	-0,838	0,038	0,009	0,045
ENSMUSG00000024654	Asrgl1	1,237	0,287	1,209	0,016	0,003	0,019
ENSMUSG00000024677	Ms4a6b	1,600	0,182	1,547	0,026	0,008	0,034
ENSMUSG00000024730	Ms4a8a	1,514	0,288	1,563	0,013	0,002	0,010
ENSMUSG00000024803	Ankrd1	2,220	0,133	2,055	0,047	0,010	0,069

ENSMUSG00000024846	Cst6	1,613	0,205	1,545	0,018	0,006	0,029
ENSMUSG00000025204	Ndufb8	1,437	0,223	1,374	0,022	0,005	0,027
ENSMUSG00000025409	Mbd6	-0,888	-0,243	-0,853	0,017	0,007	0,024
ENSMUSG00000025610	Map3k7cl	2,096	0,199	2,012	0,022	0,004	0,032
ENSMUSG00000025780	Itih5	-1,545	-0,183	-1,385	0,028	0,008	0,051
ENSMUSG00000025804	Ccr1	1,822	0,177	1,796	0,029	0,007	0,044
ENSMUSG00000025934	Gsta3	-1,437	-0,175	-1,449	0,035	0,010	0,037
ENSMUSG00000025964	Adam23	-2,348	-0,188	-1,791	0,022	0,003	0,142
ENSMUSG00000026135	Zfp142	-0,570	-0,252	-0,560	0,041	0,007	0,043
ENSMUSG00000026202	Tuba4a	1,642	0,176	1,404	0,024	0,010	0,039
ENSMUSG00000026271	Gpr35	0,973	0,211	1,074	0,038	0,010	0,026
ENSMUSG00000026489	Coq8a	1,688	0,229	1,593	0,017	0,003	0,019
ENSMUSG00000026568	Mpc2	0,947	0,281	0,945	0,014	0,003	0,014
ENSMUSG00000026994	Galnt3	-1,536	-0,222	-1,556	0,047	0,003	0,057
ENSMUSG00000027257	Pacsin3	1,577	0,218	1,278	0,030	0,004	0,065
ENSMUSG00000027329	Spef1	-0,861	-0,226	-0,847	0,018	0,009	0,019
ENSMUSG00000027384	Ndufaf5	1,325	0,264	1,282	0,011	0,004	0,017
ENSMUSG00000028273	Pdlim5	1,360	0,198	1,197	0,050	0,004	0,069
ENSMUSG00000028369	Svep1	-1,353	-0,238	-1,273	0,017	0,005	0,030
ENSMUSG00000028496	Mllt3	1,702	0,259	1,531	0,022	0,001	0,033
ENSMUSG00000028842	Ago3	0,776	0,194	0,716	0,060	0,005	0,077
ENSMUSG00000028965	Tnfrsf9	2,075	0,303	2,332	0,013	0,001	0,008
ENSMUSG00000029102	Hgfac	-2,289	-0,170	-2,590	0,052	0,005	0,046
ENSMUSG00000029156	Sgcb	1,340	0,237	1,259	0,014	0,005	0,018
ENSMUSG00000029309	Sparcl1	-1,948	-0,343	-1,698	0,013	0,001	0,025
ENSMUSG00000029442	Wdr66	1,572	0,706	1,512	0,010	0,000	0,014
ENSMUSG00000029524	Sirt4	-1,086	-0,260	-1,080	0,030	0,003	0,030
ENSMUSG00000029553	Tfec	1,419	0,194	1,231	0,023	0,009	0,053
ENSMUSG00000029594	Rbm19	0,566	0,319	0,587	0,044	0,002	0,038
ENSMUSG00000029687	Ezh2	0,675	0,236	0,656	0,031	0,006	0,037
ENSMUSG00000029761	Cald1	-0,573	-0,280	-0,576	0,080	0,004	0,046
ENSMUSG00000029769	Ccdc136	0,959	0,520	0,921	0,020	0,000	0,025
ENSMUSG00000030020	Prickle2	-0,864	-0,323	-0,855	0,016	0,002	0,018
ENSMUSG00000030142	Clec4e	2,154	0,193	1,802	0,012	0,006	0,059
ENSMUSG00000030144	Clec4d	2,220	0,202	2,189	0,024	0,003	0,046
ENSMUSG00000030187	Klra2	1,874	0,295	2,192	0,014	0,002	0,003
ENSMUSG00000030217	Art4	-1,995	-0,154	-1,875	0,035	0,008	0,068
ENSMUSG00000030329	Pianp	-1,270	-0,261	-1,255	0,022	0,003	0,022
ENSMUSG00000030680	Pagr1a	2,491	0,210	2,584	0,014	0,005	0,010
ENSMUSG00000031137	Fgf13	2,185	0,134	2,446	0,037	0,009	0,021
ENSMUSG00000031231	Cox7b	1,165	0,241	1,100	0,020	0,006	0,024
ENSMUSG00000031480	Thsd1	-0,948	-0,228	-0,946	0,022	0,008	0,024
ENSMUSG00000031494	Cd209a	-0,818	-0,230	-0,820	0,045	0,008	0,037
ENSMUSG00000031543	Ank1	1,969	0,159	1,825	0,035	0,007	0,039
ENSMUSG00000031613	Hpgd	-1,286	-0,303	-1,162	0,011	0,002	0,019
ENSMUSG00000031637	Lrp2bp	2,234	0,194	2,285	0,014	0,004	0,012
ENSMUSG00000031661	Nkd1	-0,955	-0,224	-0,967	0,016	0,010	0,015
ENSMUSG00000031712	Il15	1,133	0,295	1,093	0,020	0,002	0,023
ENSMUSG00000031748	Gnao1	-1,769	-0,234	-1,485	0,017	0,003	0,082
ENSMUSG00000031755	Bbs2	-1,008	-0,246	-1,010	0,029	0,006	0,027
ENSMUSG00000032135	Mcam	-0,718	-0,315	-0,712	0,012	0,002	0,012
ENSMUSG00000032526	Ss18l2	1,316	0,220	1,265	0,015	0,006	0,023
ENSMUSG00000032548	Slco2a1	-1,877	-0,213	-1,679	0,027	0,004	0,041
ENSMUSG00000032572	Col6a4	0,931	0,320	0,765	0,116	0,001	0,178
ENSMUSG00000032892	Rangrf	1,038	0,251	1,010	0,011	0,005	0,013
ENSMUSG00000032915	Adgre4	1,430	0,333	1,452	0,011	0,002	0,009
ENSMUSG00000033213	AA467197	1,849	0,230	2,135	0,019	0,003	0,009
ENSMUSG00000033508	Asprv1	2,999	0,138	2,492	0,042	0,004	0,112
ENSMUSG00000033845	Mrpl15	0,935	0,225	0,914	0,018	0,009	0,022

ENSMUSG00000034471	Caskin2	-0,792	-0,265	-0,794	0,012	0,005	0,012
ENSMUSG00000034652	Cd300a	1,236	0,319	1,272	0,012	0,002	0,012
ENSMUSG00000035273	Hpse	0,657	0,240	0,650	0,013	0,009	0,013
ENSMUSG00000035275	Raver2	-1,551	-0,188	-1,407	0,025	0,008	0,056
ENSMUSG00000035578	Iqcg	1,433	0,288	1,464	0,017	0,001	0,014
ENSMUSG00000035783	Acta2	-1,271	-0,327	-1,214	0,018	0,002	0,016
ENSMUSG00000035829	Ppp1r26	-2,233	-0,130	-2,021	0,045	0,007	0,083
ENSMUSG00000035877	Zhx3	-0,588	-0,275	-0,583	0,021	0,004	0,021
ENSMUSG00000036437	Npy1r	-2,180	-0,157	-1,888	0,036	0,006	0,114
ENSMUSG00000036560	Lgi4	-0,818	-0,296	-0,770	0,075	0,003	0,093
ENSMUSG00000036875	Dna2	1,026	0,226	1,025	0,045	0,005	0,044
ENSMUSG00000037031	Tspan15	-1,469	-0,300	-1,391	0,026	0,002	0,041
ENSMUSG00000037112	Sik2	-1,167	-0,206	-1,133	0,045	0,005	0,056
ENSMUSG00000037736	Limch1	1,740	0,212	1,534	0,026	0,004	0,045
ENSMUSG00000037876	Jmjd1c	-1,055	-0,361	-0,999	0,019	0,001	0,024
ENSMUSG00000038028	Tigar	1,274	0,199	1,163	0,028	0,008	0,041
ENSMUSG00000038126	Mphosph9	-1,291	-0,189	-1,158	0,059	0,005	0,106
ENSMUSG00000038147	Cd84	1,306	0,294	1,297	0,013	0,002	0,014
ENSMUSG00000038296	Galnt18	-1,264	-0,236	-1,238	0,016	0,005	0,022
ENSMUSG00000038370	Pcp4l1	-2,068	-0,142	-1,972	0,046	0,009	0,068
ENSMUSG00000038700	Hoxb5	-1,927	-0,157	-1,851	0,027	0,008	0,040
ENSMUSG00000039103	Nexn	1,811	0,168	1,581	0,032	0,009	0,047
ENSMUSG00000039903	Eva1c	-1,676	-0,253	-1,585	0,024	0,003	0,040
ENSMUSG00000039987	Phtf2	1,536	0,235	1,590	0,024	0,003	0,019
ENSMUSG00000040146	Rgl3	-1,385	-0,206	-1,419	0,027	0,007	0,026
ENSMUSG00000040729	Cep126	-0,939	-0,206	-0,986	0,074	0,009	0,053
ENSMUSG00000041073	Nacad	0,871	0,175	0,873	0,087	0,006	0,079
ENSMUSG00000041731	Pgm5	-2,864	-0,437	-2,471	0,014	0,000	0,041
ENSMUSG00000041992	Rapgef5	-0,961	-0,237	-0,984	0,024	0,006	0,020
ENSMUSG00000042207	Kdm5b	-1,212	-0,250	-1,267	0,026	0,004	0,021
ENSMUSG00000042408	Zmym6	-0,911	-0,277	-0,926	0,026	0,004	0,023
ENSMUSG00000042744	Hectd4	-1,080	-0,261	-1,009	0,019	0,004	0,030
ENSMUSG00000043004	Gng2	0,651	0,244	0,660	0,038	0,007	0,037
ENSMUSG00000043668	Tox3	-3,029	-0,103	-3,529	0,037	0,008	0,038
ENSMUSG00000044162	Tnip3	1,505	0,190	1,619	0,024	0,008	0,017
ENSMUSG00000044345	Marveld1	-0,682	-0,235	-0,684	0,013	0,010	0,011
ENSMUSG00000045761	Togaram2	2,219	0,258	1,979	0,011	0,002	0,021
ENSMUSG00000046207	Pik3r6	1,196	0,350	1,137	0,027	0,001	0,032
ENSMUSG00000046603	Tcaim	0,798	0,242	0,775	0,028	0,008	0,030
ENSMUSG00000046807	Lrrc75b	-1,397	-0,242	-1,428	0,020	0,004	0,018
ENSMUSG00000046908	Ltb4r1	1,545	0,279	1,545	0,015	0,002	0,013
ENSMUSG00000047146	Tet1	-1,516	-0,258	-1,476	0,020	0,002	0,023
ENSMUSG00000047798	Cd300lf	1,488	0,187	1,533	0,030	0,008	0,027
ENSMUSG00000048096	Lmod1	-1,949	-0,260	-1,821	0,017	0,002	0,032
ENSMUSG00000048439	Nupl2	-0,503	-0,243	-0,498	0,115	0,008	0,101
ENSMUSG00000048583	Igf2	4,687	0,627	2,653	0,021	0,000	0,141
ENSMUSG00000049643	2310022A10Ri	1,093	0,207	0,999	0,028	0,008	0,041
ENSMUSG00000051367	Six1	1,385	0,190	1,706	0,048	0,009	0,015
ENSMUSG00000053063	Clec12a	1,331	0,221	1,412	0,019	0,007	0,016
ENSMUSG00000053965	Pde5a	-2,057	-0,142	-2,047	0,049	0,009	0,067
ENSMUSG00000054203	Ifi205	1,321	0,229	1,280	0,013	0,006	0,019
ENSMUSG00000055150	Zfp78	-0,898	-0,787	-0,879	0,058	0,000	0,054
ENSMUSG00000055447	Cd47	0,852	0,366	0,837	0,017	0,001	0,015
ENSMUSG00000057469	E2f6	1,155	0,231	1,102	0,012	0,007	0,015
ENSMUSG00000058818	Pirb	1,222	0,214	1,205	0,017	0,008	0,019
ENSMUSG00000059089	Fcgr4	1,694	0,263	1,866	0,012	0,003	0,006
ENSMUSG00000059430	Actg2	-2,990	-0,325	-3,289	0,020	0,000	0,022
ENSMUSG00000059498	Fcgr3	1,540	0,182	1,588	0,026	0,009	0,022
ENSMUSG00000062169	Cnih4	1,190	0,197	1,080	0,035	0,007	0,058

ENSMUSG00000063229	Ldha	1,487	0,186	1,420	0,032	0,009	0,031
ENSMUSG00000064339	mt-Rnr2	1,175	0,250	1,193	0,027	0,005	0,019
ENSMUSG00000066440	Zfyve26	-0,895	-0,284	-0,863	0,013	0,003	0,018
ENSMUSG00000067818	Myl9	-1,758	-0,257	-1,691	0,020	0,002	0,028
ENSMUSG00000067889	Sptbn2	-2,190	-0,165	-2,362	0,032	0,006	0,033
ENSMUSG00000068270	Shroom4	-1,038	-0,313	-1,069	0,021	0,002	0,018
ENSMUSG00000068966	Zbtb34	-0,709	-0,285	-0,736	0,040	0,003	0,029
ENSMUSG00000069516	Lyz2	1,798	0,348	1,749	0,017	0,001	0,014
ENSMUSG00000069792	Wfdc17	2,268	0,217	2,109	0,024	0,002	0,039
ENSMUSG00000070047	Fat1	-0,817	-0,235	-0,825	0,017	0,010	0,015
ENSMUSG00000070565	Rasal2	-0,614	-0,314	-0,617	0,017	0,002	0,016
ENSMUSG00000072258	Taf1a	-1,095	-0,217	-1,038	0,024	0,008	0,038
ENSMUSG00000073436	Eme2	-0,958	-0,246	-0,886	0,029	0,005	0,056
ENSMUSG00000074218	Cox7a1	2,511	0,146	3,285	0,039	0,006	0,009
ENSMUSG00000075144	Olfr1156	2,035	0,221	1,991	0,012	0,003	0,013
ENSMUSG00000078566	Bnip3	0,896	0,244	0,901	0,011	0,007	0,009
ENSMUSG00000078954	Arhgap8	0,407	0,376	0,395	0,274	0,001	0,271
ENSMUSG00000079227	Ccr5	1,770	0,336	1,934	0,014	0,001	0,009
ENSMUSG00000079610	Ankrd39	0,671	0,241	0,622	0,058	0,007	0,081
ENSMUSG00000081752	Sms-ps	1,337	0,239	1,305	0,013	0,005	0,017
ENSMUSG00000083844	Ube2d-ps	1,108	0,228	0,975	0,019	0,005	0,045
ENSMUSG00000089929	Bcl2a1b	1,638	0,203	1,667	0,021	0,006	0,021
ENSMUSG00000089940	Gm4117	1,923	0,146	2,396	0,045	0,003	0,019
ENSMUSG00000095028	Sirpb1b	1,998	0,169	2,079	0,027	0,007	0,030
ENSMUSG00000096727	Psmb9	0,823	0,261	0,860	0,019	0,005	0,015
ENSMUSG00000097352	C920009B18Ri	1,328	0,179	1,327	0,034	0,009	0,033
ENSMUSG00000097797	Gm26901	1,611	0,156	1,663	0,071	0,005	0,082
ENSMUSG00000107176	Gm9794	-1,127	-0,234	-1,135	0,016	0,006	0,017
ENSMUSG00000109196	Gm44715	2,015	0,247	1,915	0,014	0,002	0,017
ENSMUSG00000112023	Lilr4b	1,348	0,189	1,264	0,041	0,010	0,068
ENSMUSG00000112280	A830082N09R	1,502	0,276	1,340	0,019	0,001	0,038
ENSMUSG00000114608	Gm36161	1,590	0,246	1,688	0,017	0,004	0,012
ENSMUSG00000026177	Slc11a1	1,052	0,184	1,107	0,010	0,017	0,007
ENSMUSG00000027077	Smtnl1	2,634	0,016	7,641	0,220	0,311	0,009
